# Impact and Modification of the New PJI-TNM Classification for Periprosthetic Joint Infections

**DOI:** 10.3390/jcm12041262

**Published:** 2023-02-05

**Authors:** Andre Lunz, Burkhard Lehner, Moritz N. Voss, Kevin Knappe, Sebastian Jaeger, Moritz M. Innmann, Tobias Renkawitz, Georg W. Omlor

**Affiliations:** 1 Department of Orthopaedics, Heidelberg University Hospital, Schlierbacher Landstrasse 200a, 69118 Heidelberg, Germany; 2 Center for Orthopedics and Joint Replacement, Marienhaus Hospital St. Wendel—Ottweiler, Am Hirschberg 1, 66606 St. Wendel, Germany

**Keywords:** periprosthetic joint infection, two-stage revision, septic surgery, total knee arthroplasty, revision arthroplasty, antibiotic-loaded bone cement spacer, PJI classification, TNM

## Abstract

The comprehensive “PJI-TNM classification” for the description of periprosthetic joint infections (PJI) was introduced in 2020. Its structure is based on the well-known oncological TNM classification to appreciate the complexity, severity, and diversity of PJIs. The main goal of this study is to implement the new PJI-TNM classification into the clinical setting to determine its therapeutic and prognostic value and suggest modifications to further improve the classification for clinical routine use. A retrospective cohort study was conducted at our institution between 2017 and 2020. A total of 80 consecutive patients treated with a two-stage revision for periprosthetic knee joint infection were included. We retrospectively assessed correlations between patients’ preoperative PJI-TNM classification and their therapy and outcome and identified several statistically significant correlations for both classifications, the original and our modified version. We have demonstrated that both classifications provide reliable predictions already at the time of diagnosis regarding the invasiveness of surgery (duration of surgery, blood and bone loss during surgery), likelihood of reimplantation, and patient mortality during the first 12 months after diagnosis. Orthopedic surgeons can use the classification system preoperatively as an objective and comprehensive tool for therapeutic decisions and patient information (informed consent). In the future, comparisons between different treatment options for truly similar preoperative baseline situations can be obtained for the first time. Clinicians and researchers should be familiar with the new PJI-TNM classification and start implementing it into their routine practice. Our adjusted and simplified version (“PJI-pTNM”) might be a more convenient alternative for the clinical setting.

## 1. Introduction

A periprosthetic joint infection (PJI) is probably the most feared complication after total joint replacement [1]. Despite significant improvements in preoperative infection prophylaxis, less invasive surgical techniques, silver-coated implants, and optimized postoperative care, the overall incidence of 0.5%–2% in primary total hip and knee replacement has not significantly decreased in recent years [2,3,4]. Two-stage revision is considered the most effective and therefore preferred treatment regimen for most chronically infected arthroplasties. However, therapeutic options include suppressive antibiotic therapy, surgical debridement with retention of implants (DAIR), one-stage septic revision, two-stage septic revision, or even definitive resection arthroplasty or amputation. The individual decision for one of the mentioned therapeutic regimens is complex and influenced by many factors [5,6,7,8,9]. Prognosis and mortality vary significantly depending on the individual preoperative situation with an overall mortality rate comparable to that of malignant diseases in the first years after diagnosis [10]. Despite the great complexity and heterogeneity of PJIs, they are mostly only classified into acute and chronic infections in the clinical setting. However, to appreciate the wide variety of preoperative situations and hereby improve evidence-based approaches and prognostic estimations, a far more comprehensive classification system is needed. In 2020 the “PJI-TNM classification” was introduced by Alt et al. to appreciate the seriousness and complexity of PJIs by using the principles of the well-known oncological TNM classification for the description of PJIs [11]. The infected implant and preoperative periarticular soft tissue situation (T = tissue and implant), the causative microorganism (N = non-human cell), and the host (M = comorbidity of the patient) were defined as the key characteristics of a PJI and transferred to the three-letter backbone of TNM [11,12]. Inspired by the oncological TNM classification, the aim of the PJI-TNM classification was to describe and assess every case thoroughly and therefore allow comparisons between different treatment options and provide a reliable tool for therapeutic recommendations and prognostic estimations already at the time of diagnosis. To the best of our knowledge, there is still very limited experience with the PJI-TNM classification even in major specialized arthroplasty centers. Therefore, the main goal of the current study is to evaluate the implementation of the new PJI-TNM classification in a clinical setting, determine its therapeutic and prognostic value and finally, if applicable, make suggestions to further improve the new classification for clinical routine use.

## 2. Materials and Methods

### 2.1. Study Population and Treatment Regimen

We conducted a retrospective cohort study including all patients with a chronic periprosthetic joint infection of the knee who were treated with a two-stage revision approach at our institution between 2017 and 2020. Approval was obtained from the local Medical Ethics Committee (S-065/2021). All included patients were diagnosed with a chronic PJI according to either the Musculoskeletal Infection Society [13] or the International Consensus Meeting [14]. Both stage-one and stage-two surgery were performed at our institution by specialized surgeons. If possible, a joint aspiration for synovial testing was performed before first-stage surgery. Furthermore, multiple tissue samples were collected intraoperatively to identify causative agents at both stages. Based on an individual intraoperative decision, either an articulating or a static spacer was implanted for the interim period. In accordance with recent literature, antibiotic treatment was usually continued until re-implantation and a second joint aspiration was not performed routinely prior to reimplantation [15,16,17].

### 2.2. The PJI-TNM Classification

Based on medical records from our institutional arthroplasty registry, all patients were retrospectively classified according to the PJI-TNM classification. The classification was inspired by and is based on the principles of the oncological TNM classification and uses the three letters T, N, and M, as its backbone [11]. “T” stands for “tissue and implant” and evaluates the preoperative soft tissue and implant situation. The causative agent and biofilm formation are represented by “N” as “non-human cells” (=bacteria and/or fungi). Preexisting other “morbidities” are addressed by “M” and categorized according to the modified Charlson Comorbidity Index [18,19]. Each item is further subclassified by a number (0–3) and a letter (a–c) to differentiate between a wide range of different situations. In case of recurrent infection of the same joint, a lower case “r” is put in front of the TNM letters [11,12]. Table 1 summarizes the original PJI-TNM classification as introduced by Alt et al. in 2020 [11].

### 2.3. Therapy and Outcome Parameters

All patients were classified according to the PJI-TNM classification. Then, relevant parameters regarding therapy and outcome (Table 2) were collected based on our prospectively organized institutional arthroplasty registry. To assess its therapeutic and prognostic value, we looked for correlations between the preoperative PJI-TNM classification and these parameters.

### 2.4. Statistical Analysis

Descriptive statistics are presented as numbers of occurrence, percentage or arithmetic mean, and standard deviation (SD). Prior to further analysis, normal distribution of the data was evaluated using a Shapiro–Wilk test. To assess association between the classification and outcome parameters, the Spearman’s rank correlation and chi-square test were conducted. In case of a statistically significant association, the effect size was calculated by determining Spearman’s rho^®^, phi-coefficient (φ) or Cramér’s V (V). The effect size is defined as small (0.1), medium (0.3), or large (0.5). The level of significance was set at *p* < 0.05 for all statistical tests and the statistical analyses were performed using SPSS (version 27.0; IBM, Armonk, New York, USA).

## 3. Results

### 3.1. The PJI-TNM Classification

During the four-year period (2017–2020), 80 patients, 40 men and 40 women, were diagnosed with chronic PJI of the knee and treated with a two-stage revision at our institution. No patients were lost to follow-up. The application of the PJI-TNM classification was possible in all cases. At the time of diagnosis, the mean age was 69 years. All patients were diagnosed with a chronic PJI and are therefore assumed to have a matured biofilm formation (no cases of “N0” in our patient cohort). Furthermore, we retrospectively classified no cases as “M3” (=patient does not survive surgical treatment) as all patients were recommended to proceed to surgery and underwent at least stage-one surgery. A total of 36 (45%) patients had a prior history of septic revision surgery of the same joint (positive r-status). A relevant preoperative soft tissue defect or fistula (T2) was present in 18 (23%) patients. Of the remaining 62 patients, a loosened implant (T1) was intraoperatively noted in 33 patients and a well-fixed implant (T0) in 29 patients. The causative agent was not identified (N1b) in 25 (31%) cases. A total of 29 (36%) cases were caused by a “non-difficult-to-treat” (N1a) organism. A total of 26 (33%) cases were classified as “N2”, 18 (23%) because of a “difficult to treat” (=N2a) and 8 (10%) because of a “polymicrobial” (=N2b) infection. No fungus infections (N2c) were identified. Every second patient had relevant comorbidities with 29 (36%) patients classified as “M1” and 11 (14%) as “M2”.

### 3.2. Therapy and Outcome Parameters

A total of 8 (10%) patients had died prior to second-stage surgery. A static spacer was used in 45 (56%) patients while an articulating spacer was used in 35 (44%) patients. The mean interim period between stage-one and stage-two surgery was 90 days (median 87 days, range 22–201 days, SD 45 days). Reimplantation was performed in 65 (81%) patients. The mean operating time of first-stage stage surgery was 190 min (median 167 min, range 76–451 min, SD 73 min) with a mean blood loss of 1070 mL (median 1000 mL, range 100–3500 mL, SD 685 mL). The extent of the bone loss was assessed according to Anderson Orthopaedic Research Institute [20]. Severe bone loss, classified as AORI 2B and 3, was present in 52 (65%) patients. In total, 8 (10%) patients underwent at least one unplanned revision surgery during the interim period, with 6 (8%) patients undergoing an exchange of the spacer and 2 (2%) patients undergoing a revision of their surgical wound. The mean length of hospital stay as an inpatient after first-stage surgery was 18 days (median 16 days, range 5–45 days, SD 9 days).

### 3.3. Correlation between “PJI-TNM” and Therapy/Outcome Parameters

The Spearman’s rank correlation and chi-square test were conducted to assess correlations between the preoperative PJI-TNM classification and therapy/outcome parameters. In the case of a statistically significant association, the effect size was calculated by determining Spearman’s rho (r), phi-coefficient (φ), or Cramér’s V (V). The effect size is defined as small (0.1), medium (0.3), or large (0.5). A prior history of septic revision surgery of the same joint (“r-status”) showed a statistically significant correlation with a medium effect size with the type of spacer used (*p* < 0.01; φ = 0.29), bone loss (*p* < 0.01, r = 0.32), and duration of first-stage surgery (*p* < 0.01, r = 0.43). There was a statistically significant association with a medium effect size between soft tissue and implant condition (“T-status”) and probability of reimplantation (*p* = 0.04, V = 0.28), as well as blood loss (*p* = 0.04, r = 0.24) and bone loss (*p* = 0.02, r = 0.26) at first-stage surgery. The comorbidities were preoperatively assessed by the Charlson Comorbidity Index (M-status) and there was a statistically significant association with a medium effect size between preoperative M-status and mortality (*p* < 0.01, V = 0.37). The results are summarized in Table 3.

### 3.4. Modification of the Original Classification Resulting in “PJI-pTNM”

**Figure 1:**
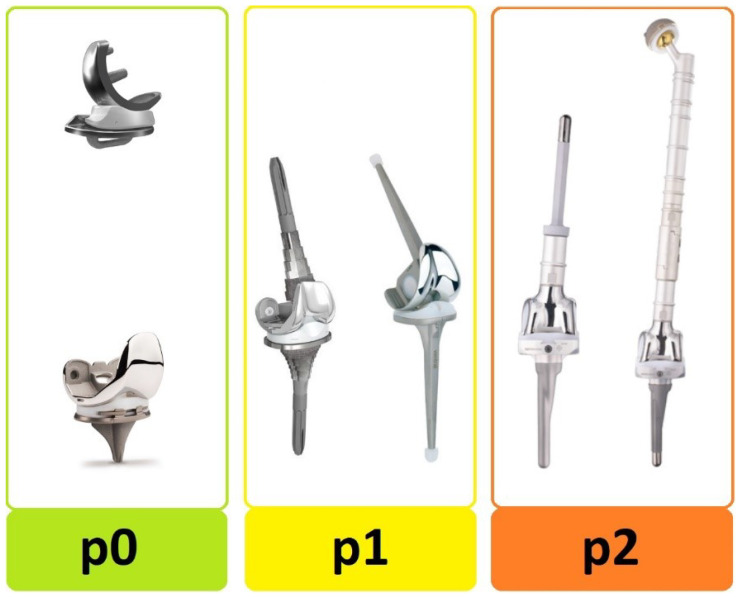
Type of infected prosthesis at time of diagnosis. The “p-status” distinguishes between: p0 = standard implant, p1 = revision implant, and p2 = megaprosthesis.

**Figure 2:**
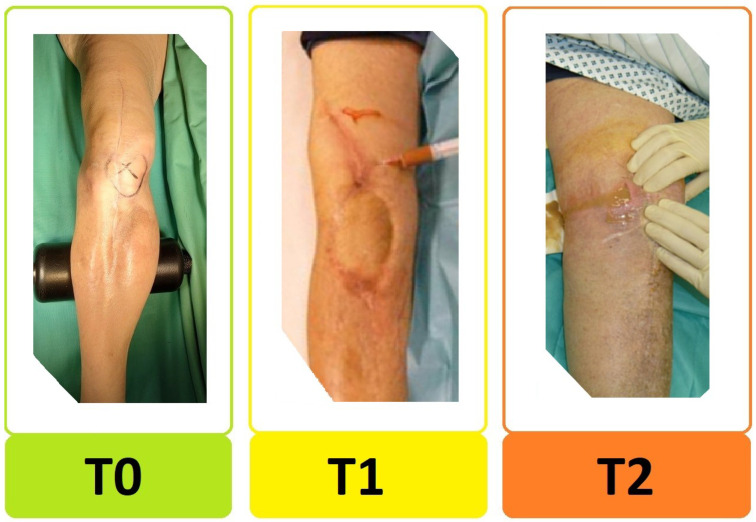
Preoperative periarticular soft tissue condition. The new “T-status” distinguishes between: T0 = good soft tissue condition, T1 = bad soft tissue condition, and T2 = periarticular fistula or significant soft tissue defect requiring flap/plastic surgery.

**Figure 3:**
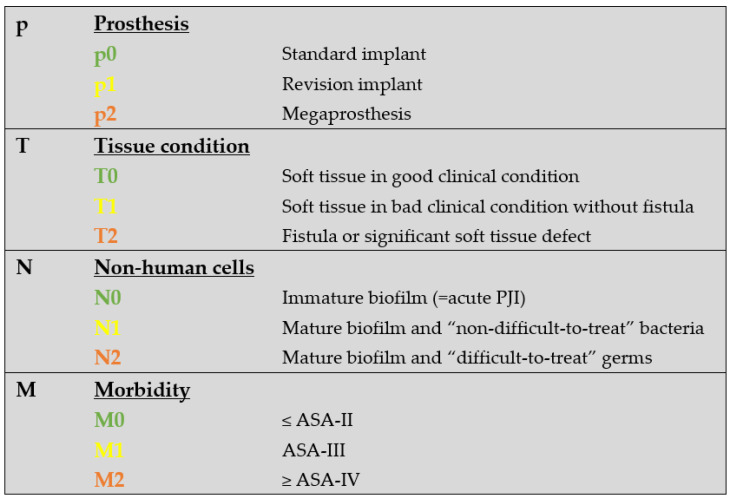
The modified PJI-pTNM classification. Type of infected prosthesis “p-status” is added to the TNM-backbone, making it “pTNM”. An “x” in front of the p-status indicates if a loosened implant is assumed or confirmed. Morbidity is assessed based on the ASA classification. The number behind each letter indicates uniformly the degree of severity on a scale from 0 to 2, with 2 being most severe. If the current PJI involves a previously infected joint, the situation is considered as “reinfection” and an “r” is put in front of pTNM.

**Figure 4:**
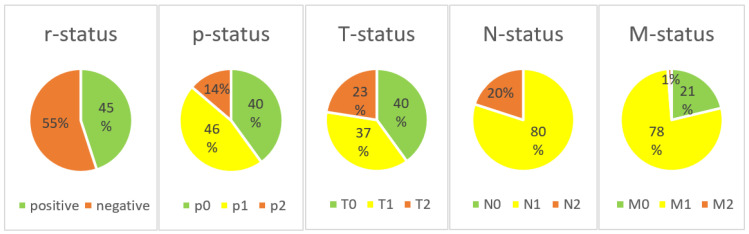
Distribution of subtypes according to the modified PJI-pTNM classification in our patient cohort.

We have modified the original classification to simplify it for clinical use. First, the type of the infected prosthesis “p-status” (Figure 1) was added to the TNM-backbone making it “pTNM”. To indicate a loosened implant an “x” was put in front of the “p-status”. Then, we simplified the general structure by uniformly indicating the degree of severity by a three-step system: 0 = least serious, 1 = moderate, and 2 = most serious. By separating implant and tissue condition (original “T-status”), the new “T-status” (Figure 2) represents strictly and only the soft “tissue situation” while the new p-status represents the implant situation. The “N-status” still represents the causative agent (or “non-human cells”) but was simplified to differentiate between 3 (instead of originally 7) different situations. Furthermore, we suggest assessing morbidity (“M-status”) based on the American Society of Anesthesiologists Physical Status Classification System (ASA) [21] instead of Charlson Comorbidity Index [18]. The modified PJI-pTNM classification is summarized in Figure 3 and an overview of the 80 cases according to the modified version is given in Figure 4.

### 3.5. Correlation between Modified PJI-pTNM and Therapy/Outcome Parameters

We found a statistically significant correlation with a large effect size between the type of infected prosthesis (“*p*-status”) and the following two characteristics: type of spacer used for the interim period (*p* < 0.01, φ = 0.62) and bone loss during first-stage surgery (*p* < 0.01, r = 0.69). Furthermore, there was a statistically significant association with a medium effect size between *p*-status and blood loss (*p* < 0.01, r = 0.36) at first-stage surgery. The preoperative soft tissue condition (new “T-status”) was associated (all medium effect size) with the probability to proceed to reimplantation (*p* = 0.01, φ = 0.28), type of spacer used for the interim period (*p* = 0.04, φ = 0.23), duration of the interim period (*p* = 0.045, r = 0.25), bone loss (*p* = 0.04, r = 0.23) and blood loss (*p* < 0.01, r = 0.32) at first-stage surgery. The patient’s preoperative comorbidity was assessed by an anesthesiologist according to the ASA classification (new “M-status”). A statistically significant association with a medium effect size was identified between the new M-status and mortality (*p* = 0.04, φ = 0.23). A statistically significant correlation with a medium effect size was identified between the new “N-status” and the following parameters: rate of unplanned revision surgeries during the interim period (*p* = 0.02, φ = 0.28), type of spacer used (*p* = 0.04, φ = 0.23), duration of interim period (*p* = 0.02, r = 0.29), and operating time (*p* = 0.01, r = 0.31) at first-stage surgery. The results are summarized in Table 4.

## 4. Discussion

A PJI is one of the most serious complications after total joint replacement. Despite its great complexity and enormous heterogeneity, clinicians mostly only differentiate between two cases, an acute or a chronic infection. This differentiation is clinically important and relevant, as an acute PJI can be treated without removing the implants with the so-called DAIR procedure. However, of course, this simple classification does not appreciate the complexity and heterogeneity of PJIs. Another well-known and widely used classification was introduced by Tsykayama et al. in 1996. It distinguishes between four different scenarios: unexpectantly positive intraoperative cultures, early postoperative infection, late chronic infection, or acute hematogenous infection [22]. Again, this is a simple and clinically relevant classification as it has direct influence on the therapeutic approach. However, to improve evidence-based approaches in the challenging setting of a PJI, a more comprehensive classification system was needed. In 2020, the “PJI-TNM classification” was introduced by Alt et al. to appreciate the seriousness and complexity of PJIs, as they often result in a morbidity and mortality similar to malignant tumor diseases. This classification was inspired by and uses the principles of the well-known oncological TNM classification for the description of PJIs [11]. The oncological TNM system is clearly structured, memorable, and successfully tested in complex and heterogenous situations in the setting of different cancers. The PJI-TNM classification uses the three-letter backbone of TNM to describe the following characteristics of a PJI: T = infected implant and periarticular soft tissue situation, N = causative microorganism, and M = comorbidity of the patient [11,12]. To the best of our knowledge, there are no reports on the classification’s feasibility and implementation in a clinical setting. Therefore, the goal of this study was to evaluate the implementation of the new PJI-TNM classification in a clinical setting and determine its therapeutic and prognostic value. A total of 80 consecutive patients treated with a two-stage revision for periprosthetic knee joint infection were included in this study. Based on data from our institutional arthroplasty registry, we were able to retrospectively classify all our 80 patients resulting in 52 different cases according to the original PJI-TNM classification. Although our patient cohort included only chronic PJIs, the classification is generally appropriate to differentiate between acute and chronic infections by distinguishing between an immature and mature biofilm formation. In our patient cohort, we identified significant correlations between the preoperative PJI-TNM classification and relevant therapeutic and prognostic parameters. A previous infection of the same joint (r-status) correlates with bone loss, duration, and type of spacer inserted at first-stage surgery. The preoperative implant and periarticular soft tissue situation (T-status) has a significant influence on the probability of reimplantation in the setting of two-stage revision. Furthermore, it also correlates with the invasiveness (bone loss and blood loss) of first-stage surgery. The patient’s comorbidity (M-status) directly correlates with mortality. We have demonstrated that the PJI-TNM classification provides an objective tool to estimate the invasiveness of surgery, the probability to proceed to reimplantation, and even mortality during the first 12 months after diagnosis. These predictions can be made preoperatively based on the preoperative PJI-TNM classification and used to improve individual therapeutic decisions in the challenging situation of a PJI. However, because of its complexity and the enormous variety of subtypes, we believe that a simplification would be helpful to successfully implement the PJI-TNM classification into clinical routine use.

### 4.1. The Modified PJI-pTNM Classification

**Figure 5:**
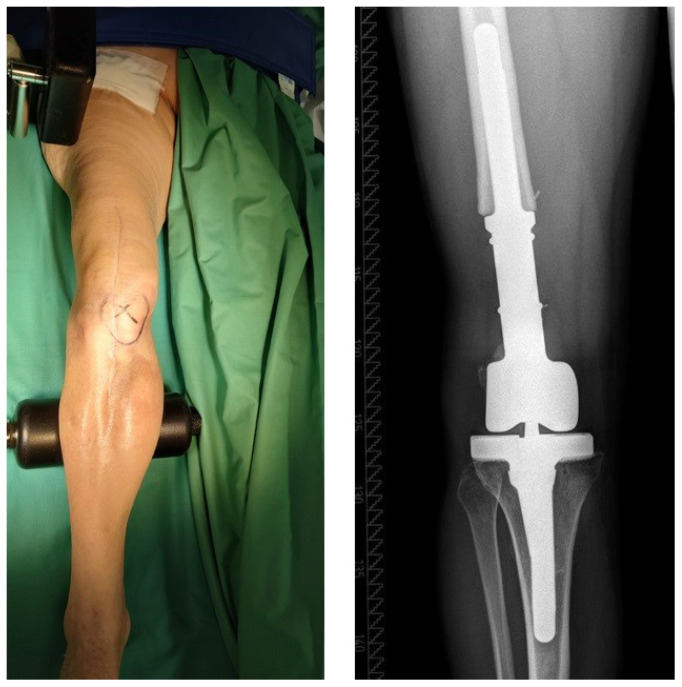
This patient was diagnosed with a chronic PJI of a megaprosthesis (“p2”) with periarticular soft tissues in a good clinical condition (“T0”). The patient underwent two-stage revision surgery with successful reimplantation of a new megaprosthesis at second-stage surgery.

**Figure 6:**
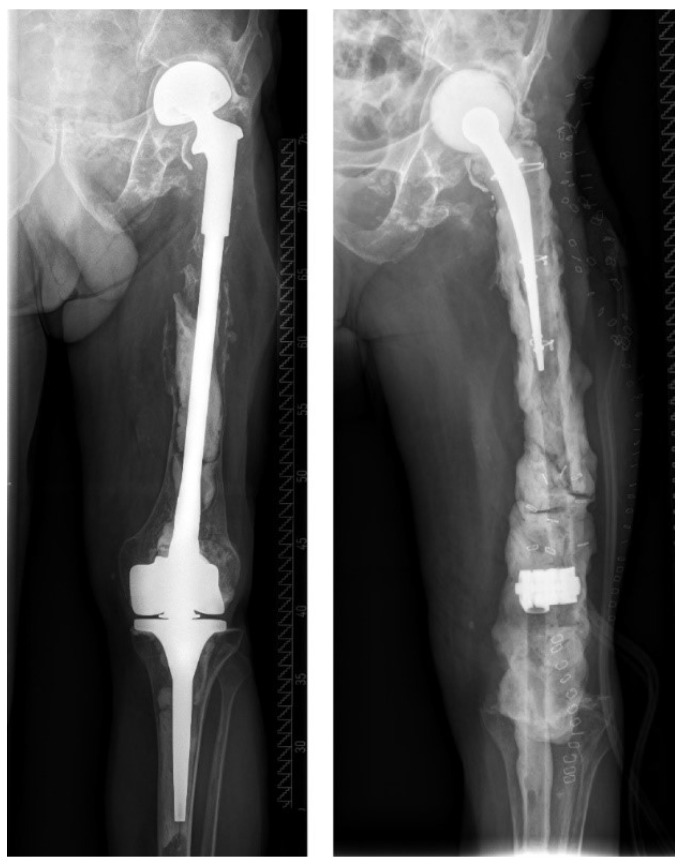
This patient was diagnosed with a chronic PJI of his total femur prosthesis (“p2”) and underwent first-stage surgery with implantation of a custom-made double-level spacer (hip and knee spacer of the same leg). Because of the patient’s high perioperative risk (“M2”) and low physical demand, a shared decision was made to not perform second-stage surgery.

We believe the original PJI-TNM classification is a great and valuable tool. It follows the principles of the well-known oncological TNM classification for the description of PJIs. However, because of its complexity and the enormous variety of subtypes with theoretically over 300 different cases distinguishable, we also believe that a modification could improve the implementation of the classification into clinical routine use. Our goal was not to introduce a new classification but to simplify the original classification without changing its structural backbone or losing the identified correlations. We tried to improve the classification’s substructure by uniformly indicating the degree of severity using a three-step system: 0 = least serious, 1 = moderate, and 2 = most serious, for each of the main characteristics of the TNM-backbone. This also significantly reduced the total number of different subtypes (Table 5). Furthermore, we introduced a fourth main characteristic, the “p-status”, to distinguish between three types of implants: a standard implant (p0), a revision implant (p1), and a megaprosthesis (p2). Our results confirmed that the type of the infected implant has a significant influence on the invasiveness of surgery and outcome. In the current study, 12 (15%) patients had the most challenging situation of a PJI involving a megaprosthesis. Figure 5 and Figure 6 show X-rays of two patients with infected megaprostheses. Our modified version clearly highlights that these are extremely challenging situations (“p2”), while the original classification fails to do so. In this study, we have demonstrated that a PJI of a megaprosthesis generally results in more invasive surgery with a higher extent of blood and bone loss as well as implantation of a static spacer for the interim period. Therefore, we recommend referring every “p2” case to specialized centers for further treatment.

The N-status represents “non-human cells” or the causative agent and was significantly simplified to differentiate only between the following three situations: N0 = immature biofilm, N1 = “non-difficult-to-treat” germs, and N2 = “difficult-to-treat” germs (no biofilm active drugs (rifampicin or ciprofloxacin) available for treatment). These three situations have a direct impact on the therapeutic approach. A PJI with an immature biofilm formation (N0) can be treated with the DAIR procedure (debridement, antibiotics, and implant retention). If a “difficult-to-treat” germ (N2) is identified, a single-stage approach is strongly discouraged as biofilm-active drugs are not available [23]. Furthermore, this study identified that PJIs caused by “difficult-to-treat” germs had to be addressed with additional exchange of the spacer significantly more often (multi-stage approach) than PJIs caused by “non-difficult-to-treat” bacteria.

For more than 50 years, anesthesiologists have been using the American Society of Anesthesiologists (ASA) Physical Status Classification System to stratify patient’s preoperative comorbid conditions and estimate the perioperative risk. Although the ASA classification is not specifically designed to identify risk factors in the setting of PJIs or even orthopedic surgery, we believe that using the ASA classification is very convenient for clinicians as it is well-known and usually provided by an anesthesiologist before surgery. Furthermore, many national arthroplasty registers routinely collect the ASA classification, making it the most widely used classification to assess morbidity in total joint replacement. Finally, our results suggest a similar prognostic value of the ASA score compared to the CCI, as both showed significant correlations with patient mortality during the first 12 months after diagnosis.

### 4.2. Limitations of the Study

This is a single-center retrospective cohort study. However, to the best of our knowledge, it is the first series evaluating the feasibility and implementation of the PJI-TNM classification in a clinical setting. The transfer of our results on PJIs of other joints may be compromised as only chronic PJIs of the knee were included in this study. However, at the same time, our strict inclusion criteria resulted in a very homogenous patient cohort, which allowed a more thorough analysis. We hope that prospective studies with longer follow-up periods and more patients will confirm our results and provide further correlations between the preoperative PJI-(p)TNM classification and prognostic parameters.

## 5. Conclusions

The PJI-TNM classification is inspired by and based on the well-known oncological TNM classification to appreciate the seriousness and complexity of PJIs. We have demonstrated that both classifications, the original and our modified version, provide reliable predictions at the time of diagnosis about the invasiveness of surgery (duration of surgery, blood and bone loss), probability to proceed to reimplantation in a two-stage approach, and patient mortality during the first 12 months after diagnosis. Orthopedic surgeons can use the new classification system preoperatively as an objective and comprehensive tool for therapeutic decisions and patient information (informed consent). Furthermore, as the new classification system takes many important characteristics into account, it allows for the first time to compare different treatment options for truly similar preoperative situations. We believe orthopedic surgeons and researchers should be familiar with the new PJI-(p)TNM classification and start implementing it into their routine practice. Especially clinicians might prefer our modified version (“PJI-pTNM”) as it was specifically adjusted and simplified for a more convenient use in the clinical setting. At our institution, we are starting to implement the modified classification into our clinical routine.

## Figures and Tables

**Table 1:** The original PJI-TNM classification as introduced by Alt et al. in 2020.

R			If the Infection Involves a Previously Infected Implant, the Situation Is Considered as “Reinfection” and an “r” Is Put in Front of the Classification
**T**	**T0**	a	Stable standard implant without important soft tissue defect
		b	Stable revision implant without important soft tissue defect
	**T1**	a	Loosened standard implant without important soft tissue defect
		b	Loosened revision implant without important soft tissue defect
	**T2**	a	Severe soft tissue defect with standard implant
		b	Severe soft tissue defect with revision implant
**N**	**N0**	a	No mature biofilm formation (former: acute), directly postoperatively
		b	No mature biofilm formation (former: acute), late hematogenous
	**N1**	a	Mature biofilm formation (former: chronic) w/o “difficult-to-treat bacteria”
		b	Mature biofilm formation (former: chronic) with culture-negative infection
	**N2**	a	Mature biofilm formation (former: chronic) with “difficult-to-treat bacteria”
		b	Mature biofilm formation (former: chronic) with polymicrobial infection
		c	Mature biofilm formation (former: chronic) with fungi
**M**	**M0**		Not or only mildly compromised (Charlson Comorbidity Index: 0–1)
	**M1**		Moderately compromised patient (Charlson Comorbidity Index: 2–3)
	**M2**		Severely compromised patient (Charlson Comorbidity Index 4–5)
	**M3**	a	Patient refuses surgical treatment
		b	Patient does not benefit from surgical treatment
		c	Patient does not survive surgical treatment

**Table 2:** Therapy and outcome parameters examined in this study.

Therapy and Outcome Parameters
Mortality (survived or died)
Type of spacer used (articulating or static)
Reimplantation at second-stage surgery (yes or no)
Unplanned revision surgery during interim period (yes or no)
First-stage surgery: duration of surgery (min), blood loss (ml), bone loss (AORI)
Duration of interim period (days)

**Table 3:** PJI-TNM classification and outcome parameters were analyzed by conducting the Spearman’s rank correlation and chi-square test. Level of significance was set at *p* < 0.05 and, if achieved, is written in bold letters and marked with an asterisk (*). In case of a statistically significant association, the effect size was calculated by determining Spearman’s rho (r), phi-coefficient (φ), or Cramér’s V (V). The effect size is defined as small (0.1), medium (0.3), or large (0.5). Bone loss was assessed according to AORI and categorized as severe (AORI 2b and 3) or not (AORI 1 and 2a).

	r-Status	T-Status	N-Status	M-Status
Mortality (yes, no)	*p* = 0.706	*p* = 0.61	*p* = 0.77	***p*** < 0.01 *; V = 0.37
Spacer (articulating, static)	***p*** < 0.01 *; φ = 0.29	*p* = 0.11	*p* = 0.18	*p* = 0.74
Reimplantation (yes, no)	*p* = 0.20	***p*** = 0.04 *; V = 0.28	*p* = 0.67	*p* = 0.63
Duration of 1st stage (min)	***p*** < 0.01 *; r = 0.43	*p* = 0.18	*p* = 0.08	*p* = 0.42
Blood loss at 1st stage (mL)	*p* = 0.09	***p*** = 0.04 *; r = 0.24	*p* = 0.15	*p* = 0.67
Bone loss at 1st stage	***p*** < 0.01 *; r = 0.32	***p*** = 0.02 *; r = 0.26	*p* = 0.65	*p* = 0.92
Spacer revision (yes, no)	*p* = 0.25	*p* = 0.95	*p* = 0.12	*p* = 0.90
Interim period (days)	*p* = 0.24	*p* = 0.29	*p* = 0.98	*p* = 0.55

**Table 4:** The “modified PJI-pTNM classification” and therapy/outcome parameters were analyzed for correlations by conducting the Spearman’s rank correlation and chi-square test. Level of significance was set at *p* < 0.05 and, if achieved, is written in bold letters and marked with an asterisk (*). In case of a statistically significant correlation, the effect size was calculated by determining Spearman’s rho (r), phi-coefficient (φ), or Cramér’s V (V). The effect size is classified as small (0.1), medium (0.3), or large (0.5).

	*p*-Status	T-Status	N-Status	M-Status
Mortality (yes, no)	*p* = 0.09	*p* = 0.33	*p* = 0.60	*p* = 0.04 *; φ = 0.23
Spacer (art., static)	***p*** < 0.01 *; φ = 0.62	***p*** ** = ** 0.04 *; φ = 0.23	***p*** = 0.04 *; φ = 0.23	***p*** = 0.03 *; **φ = 0.25**
Reimplantation (yes, no)	*p* = 0.74	***p*** ** = ** 0.01 *; φ = 0.28	*p* = 0.35	*p* = 0.10
Duration of 1st stage (min)	***p*** < 0.01 *; r = 0.37	*p* = 0.10	***p*** ** = ** 0.01 *; r = 0.31	*p* = 0.72
Blood loss at 1st stage (mL)	***p*** < 0.01 *; r = 0.36	***p*** < 0.01 *; r = 0.32	*p* = 0.58	*p* = 0.16
Bone loss at 1st stage (AORI)	***p*** < 0.01 *; r = 0.69	***p*** = 0.04 *; r = 0.23	*p* = 0.84	*p* = 0.37
Revision of spacer (yes, no)	*p* = 0.47	*p* = 0.76	***p*** = 0.02 *; φ = 0.28	*p* = 0.50
Interim period (days)	*p* = 0.37	***p*** = 0.045 *; r = 0.25	***p*** ** = ** 0.02 *; r = 0.29	*p* = 0.16

**Table 5:** Comparison of cases between original “TNM” and modified “pTNM” classification according to the 80 cases analyzed in this study.

No.	Mod. PJI-pTNM	Original PJI-TNM
1	p0T0N1M0 (4×)	T0aN1aM0, T0aN2bM0, T1aN1bM0 (2×)
2	p0T0N1M1 (12×)	T0aN1aM1, T0aN1aM2 (3×), T0aN1bM2, T0aN2bM1, T1aN1aM0, T1aN1aM1 (3×), T1aN1aM2, T1aN1bM0
3	p0T0N2M1 (3×)	T0aN2aM0, T0aN2aM1, T1aN2bM0
4	p0T1N1M0 (3×)	T0aN1bM0, T1aN1aM0, T1aN1bM0
5	p0T1N1M1 (3×)	T0aN1aM0, T0aN1bM1, T1bN1aM1
6	p0T1N2M1	T0aN2aM0
7	p0T2N1M1 (4×)	T2aN1aM1 (2×), T2aN1bM0, T2aN2bM1
8	p0T2N1M2	T2aN1bM2
9	p1T0N1M1 (6×)	T0bN1aM1 (2×), T0bN1aM2, T1bN1aM1, T1bN1bM0, T1bN1bM1
10	p1T0N2M1 (2×)	T0bN2aM0, T0bN2aM1
11	p1T1N1M0 (5×)	T0bN1bM0 (2×), T1bN1bM0 (2×), T1bN2bM0
12	p1T1N1M1 (13×)	T0bN1aM0, T0bN1bM1 (3×), T1bN1aM0, T1bN1bM0 (4×), T1bN1bM1, T1bN2bM0 (2×), T1bN2bM1
13	p1T1N2M0 (2×)	T0bN2aM0, T0bN2aM1
14	p1T1N2M1 (2×)	T0bN2bM2, T1bN2aM1
15	p1T2N1M1 (3×)	T2bN1aM1, T2bN1aM2, T2bN2bM0
16	p1T2N2M0	T2bN2aM0
17	p1T2N2M1 (4×)	T2bN2aM0, T2bN2aM1, T2bN2aM1, T2bN2bM0
18	p2T0N2M0	T0bN2aM0
19	p2T0N2M1	T1bN2aM2
20	p2T1N1M0	T1bN1aM1
21	p2T1N1M1 (3×)	T0bN1aM2, T1bN1bM0 (2×)
22	p2T2N1M0	T2bN1aM0
23	p2T2N1M1 (2×)	T2bN1aM1 (2×)
24	p2T2N1M1	T2bN1aM0
25	p2T2N2M1	T2bN2bM0

## Data Availability

The data presented in this study are available on request from the corresponding author. The data are not publicly available due to patients’ data protection.
